# Supplementary material for: A ‘resource allocator’ for transcription based on a highly fragmented T7 RNA polymerase
Source: Mol Syst Biol. 2014 Jul 30;10(7):742. doi: 10.15252/msb.20145299 (PMC4299498; doi:10.15252/msb.20145299)
Supplement: Supplementary file 1 — Supplementary Figures and Tables [file msb0010-0742-sd1.pdf]

Supplementary Information for:

A ‘resource allocator’ for transcription based on a highly fragmented T7 RNA polymerase

Thomas H. Segall-Shapiro, Adam J. Meyer, Andrew D. Ellington, Eduardo D. Sontag, and Christopher A. Voigt

**I.**	**Splitposon method for bisection mapping proteins**		**2-4**
	I.A.	*Design of the splitposon*	*2*
	I.B.	*Library generation and characterization*	*3*
**II.**	**Bisection mapping of T7 RNA polymerase**		**5-7**
	II.A.	*Library design and statistics*	*5*
	II.B.	*Library characterization*	*5*
	II.C.	*Split sites shown on the T7 RNAP structure*	*7*
**III.**	**Supporting experiments**		**8-14**
	III.A.	*Directed evolution of the K1F and N4 σ fragments*	*8*
	III.B.	*Means and error underlying the σ fragment orthogonality matrix*	*9*
	III.C.	*Identifying the null fragment*	*10*
	III.D.	*Activation of the β core fragment with proteins fused to the α fragment*	*11*
	III.E.	*Measurement of P_{Tac} activity*	*12*
	III.F.	*Growth impact of split polymerase expression*	*13*
**IV.**	**Mathematical models**		**15-21**
	IV.A.	*Kinetic model of the resource allocator*	*15*
	IV.B.	*Uniqueness and stability of steady states in resource allocator model*	*17*
	IV.C.	*Modeling σ fragment competition data*	*21*
**V.**	**Plasmid details**		**22-25**
**VI.**	**References**		**26-27**

I. Splitposon method for bisection mapping proteins

I.A. Design of the splitposon

The splitposon is based on a commercial mini-Mu transposon, the HyperMu <KAN-1> transposon (previously available from Epicentre Biotechnologies). Mini-Mu transposons are a commonly used tool in molecular biology, due to their small size and easy *in vitro* transposition protocol (Haapa *et al*, 1999). *In vitro* transposition requires only the addition of a single transposase protein, MuA, along with a linearized mini-Mu transposon. The MuA protein binds specific sequences at the termini of the transposon ('recognition ends') and catalyzes an efficient, mostly sequence-independent transposition event (Mizuuchi & Mizuuchi, 1993; Green *et al*, 2012). In contrast to the native transposon, which contains 6 unique sequences in the recognition ends (L1-L2-L3 at one terminus, R1-R2-R3 at the other), mini-Mu transposons have shorter, palindromic ends consisting of two of the native sequences (R1-R2) (Haapa *et al*, 1999).

While the R1-R2 sequence is required for transposition of a mini-Mu transposon, the sequence does not have to be perfect. The promiscuity of MuA has been studied by mutating the ends of the transposon , and a number of functional transposons with altered ends have been made. To construct the splitposon, we pooled the information from these studies to identify where the transposon could be altered and retain function. We focused on the R1 recognition sequence, since it is closest to the ends of the mini-Mu transposon, and our intention was to split proteins with as little added sequence as possible.

First, we used a consensus alignment of the six recognition sequences from the natural transposon (Goldhaber-Gordon *et al*, 2002) to determine where mutations are generally tolerated. However, it is unclear whether all of these alterations are tolerated specifically in the terminal recognition sites. Next, the R1 sequence was aligned with the L1 sequence, which is at the opposite terminus of the natural transposon. We referenced a mutational study (Lee & Harshey, 2001) to determine tolerated changes to the two bases at the end of the transposon when it is used for *in vitro* transposition reactions. Finally, we collated the mutations in previously built transposon variants. Variants with a NotI cut site insertion and a triple stop codon insertion (Poussu *et al*, 2004, 2005) have been included in commercially available kits (F-701 and F-703 from Thermo Scientific), and have high activity. In addition, transposons with two unique MlyI cut site insertions and two unique AarI cut site insertions are specified in publications (Jones, 2006; Hoeller *et al*, 2008).

A start codon was introduced into the -4 through -2 positions in the transposon. The RBS calculator (thermodynamic model v1.0) (Salis *et al*, 2009) was used to evaluate a number of potential transposon ends for strong RBS activity. One variant proved to retain sufficient transposition efficiency and effectively initiate translation at the start codon. A P_{Tac} IPTG inducible promoter system from pEXT20 (Dykxhoorn *et al*, 1996) mutated to have a symmetric LacO site ("aattgtgagcgctcacaatt") was added to the splitposon to drive expression of the C-terminal protein fragment. The constitutive LacI cassette was included so that the promoter would not drive high levels of expression when in a plasmid lacking LacI expression.

The natural mini-Mu transposon contains a stop codon in-frame with the newly engineered start codon. However, out of frame insertions can lead to many additional amino acids added to the N-terminal fragment of the split protein, potentially complicating the analysis of bisection libraries. For this reason, we mutated the terminus of the splitposon opposite from the start codon to contain three staggered stop codons (one stop codon in each frame). This modification had already been successfully made in a mini-Mu transposon end to create a transposon for generating libraries of truncated proteins (Poussu *et al*, 2005).

I.B. Library generation and characterization

The splitposon can be used to split a protein of interest with two standard cloning steps (Fig 2A). First, MuA is used to transpose the splitposon into a target insertion plasmid (Supplementary Fig S2B), which contains the region of the gene of interest to be targeted for bisection. This library is selected for the Kanamycin resistance gene in the transposon in addition to the resistance gene on the insertion plasmid. A sufficient number of colonies to achieve good coverage are plated, scraped, and harvested to yield an 'insertion library'. Second, the pooled insertion library is digested using Type IIs restriction sites flanking the region of interest. The digested library is run on a gel, and the band with size corresponding to the region of interest plus a single splitposon is excised and purified. Finally, the size-selected fragments are ligated into an expression plasmid (Supplementary Fig S2C) that has also been digested with Type IIs restriction enzymes to produce compatible overhangs. This plasmid contains an inducible expression system, as well as any flanking portions of the gene that were not in the region of interest.

The single transposition yields 6 different outcomes, depending on the orientation and position of the splitposon in the protein that is being split (Supplementary Fig S1). The splitposon can insert in either the forward or reverse direction. If it is in the reverse direction, only the N-terminal fragment of the protein is expressed, and this fragment has a number of additional bases fused to it depending on the exact insertion location. Reverse transpositions therefore, are only seen if the protein of interest can be truncated and retain function. If transposition is targeted to a region of the protein that is not sufficient for function (*i.e.*, by choosing a small enough region for the insertion plasmid), reverse insertions should have no function and will not be seen in a final selected library.

When the transposon is inserted in the forward direction, the frame of insertion determines what protein fragments will be made. MuA transposition duplicates 5 bp, leading to a few added amino acids on the protein fragments and complicating analysis. If the transposon inserts in frame with respect to the protein fragment at the 5' end of the transposon (frame 0), then a split protein will be expressed as desired. The N-terminal fragment contains no added amino acids, and the C-terminal fragment contains 3 added amino acids: M (for the start codon), a variable residue (coded for by A12, where 1 and 2 are the first two duplicated bp), and a duplicated residue (coded for by 345, the last three duplicated bases). If the transposon is inserted in frame +1 or +2, the C-terminal protein fragment is likely not to be expressed, leading to truncations that should not appear in a selected or screened library. Occasionally, the transposon may insert in frame +1 or +2 very close to an in-frame start codon, or it may create a start codon with the terminal A. In this case, out-of frame split proteins can be expressed, where the N-terminal fragment contains 2-3 variable/added residues (before the latter stop codons are encountered), and the C-terminal fragment contains duplications, insertions, or deletions based on the location of the start codon.

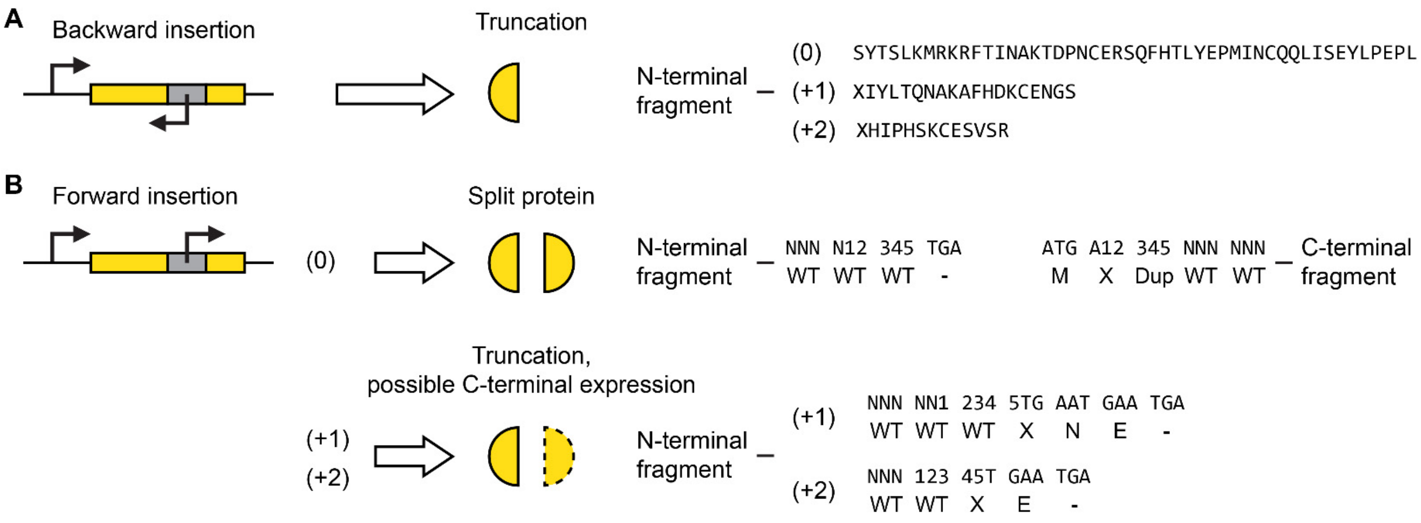

Supplementary Figure S1. Outcomes of a splitposon library. *(**A**) If the splitposon inserts in the reverse direction, only the N-terminal fragment of the protein is expressed. Additionally a number of amino acids are fused to this fragment depending on the frame of insertion (as judged by protein fragment at the 5' end of the transposon). X indicates a variable residue that depends on the sequence of the insertion site. (**B**) If the splitposon inserts in the forward direction, a split protein or truncation is expressed depending on the frame of insertion. If the splitposon inserts in-frame (0), a split protein is expressed with 3 AAs added to the C-terminal fragment. The DNA sequence and encoded AAs directly flanking the splitposon are shown. For DNA (top row), Ns indicate bases in the original coding sequence of the protein, 1-5 indicates the 5 bps of DNA duplicated during MuA transposition, and other letters indicate the sequence of the splitposon. For AAs (bottom row), WT indicates a residue in the split protein, X indicates a variable residue (i.e. one coded for by bps both from the splitposon and original protein coding sequence), Dup indicates a WT residue that is present in both the N and C-terminal fragments, and other letters represent the appropriate AAs. If the splitposon inserts in the (+1) or (+2) frames, the N-terminal fragment will be expressed with a few added AAs and the C-terminal fragment may be expressed by an in-frame start codon. The residues added to the N-terminal fragment are shown in the same manner as for the (0) frame.*

II. Bisection mapping of T7 RNA polymerase

II.A. Library design and statistics

To avoid seeing any truncations in the library of bisected T7* RNAP, we chose to target transpositions to a subset of the gene. Previous studies on T7 RNAP have identified the C-terminus of the gene as having a key role in catalysis and function. A version of the polymerase lacking the last two residues has been shown to lack productive polymerase activity (Mookhtiar *et al*, 1991). We excluded the last 7 residues of the gene from our library to ensure that functional truncations would not be generated. In contrast, the N-terminal region of the gene appears less sensitive to alterations. A pilot library indicated that truncations of up to 30-35 residues were tolerated, so we conservatively excluded the first 40 residues from our bisection library. Hence, the insertion plasmid contains only residues 41-876 of T7* RNAP (Supplementary Fig S2B). This section of the gene is flanked by BsaI Type IIs restriction sites for subcloning. We chose a ColEI backbone with Ampicillin resistance for the insertion plasmid. For the expression plasmid, the flanking portions of the polymerase (AAs 1-40 and 877-883) were placed downstream of the same P_{Tac} expression system that is in the splitposon (Supplementary Fig S2C). BsaI restriction sited are located between these fragments to allow seamless subcloning of the T7 RNAP* 41-876 fragment from the insertion plasmid. Based on the size of the insertion plasmid and T7 RNAP* fragment it contains we calculated the library sizes of the insertion and final libraries. Based on the number of colonies harvested for each library, sufficient coverage was achieved at each library step to achieve a high probability of sampling all possible variants (Supplementary Table S1) (Patrick *et al*, 2003).

II.B. Library characterization

After the final split T7* RNAP library was built and harvested, it was transformed into cells containing the plasmid Nif_489 (Temme *et al*, 2012). This plasmid contains a P_{T7} driven RFP gene. Colonies were plated on selective media and 384 visually red colonies were picked (P_{Tac} is leaky enough on plates that colonies were visibly red without IPTG induction). These colonies were assayed for fluorescence in liquid media and the most active 192 selected for sequencing and further analysis. Each of the 192 selected clones was assayed four times and the mean promoter activity calculated.

The 192 active clones were each sequenced to determine the splitposon insertion location. In 180/192 clones this sequencing read gave enough information to unambiguously determine the insertion site of the splitposon. The other 12/192 clones were double splitposon insertions, other failure modes of the library, or sequencing errors, and were discarded. Of the 180 sequenced clones, 56 unique split sites were identified, with 36 in-frame and 20 out-of frame. The vast majority of the out-of-frame splits inserted in a location predicted to have a close downstream in-frame start codon, leading to a split protein. However, due to high predicted variability in the RBS strength for out-of-frame splitposon insertions, we focused on the in-frame splits for all further analysis. Information on the 192 analyzed clones is given in the source data for Fig 2B.

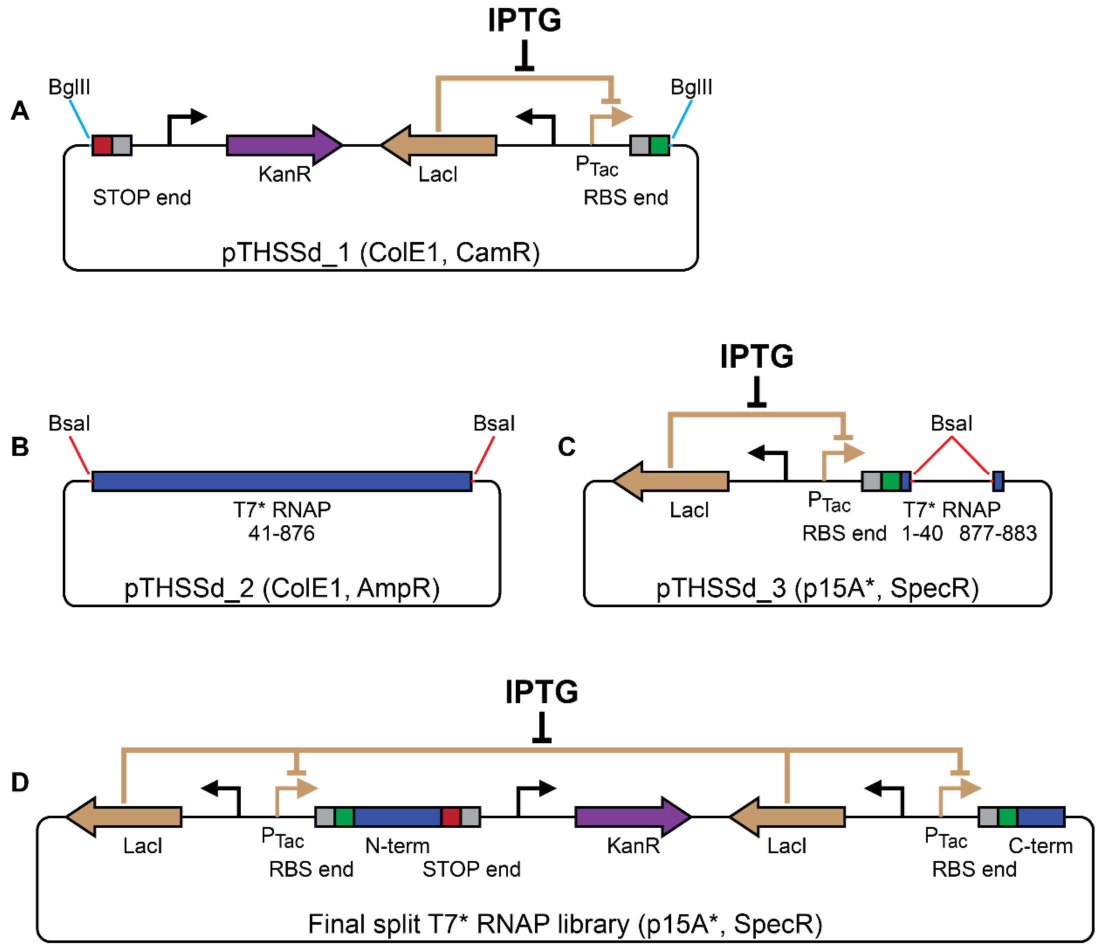

Supplementary Figure S2. Plasmids used for bisection mapping of T7 RNA polymerase. *(**A**) The splitposon is carried in a high copy ColE1 plasmid with chloramphenicol resistance. It is excised with BglII and purified from an agarose gel to produce the 'cleaved' linear transposon substrate for an* in vitro *transposition reaction. (**B**) The insertion plasmid carries the coding sequence for residues 41-876 of T7* RNAP flanked by BsaI sites on a high copy ColE1 backbone with ampicillin resistance. (**C**) The expression plasmid contains an inducible P_{Tac} expression system and the coding sequences for residues 1-40 and 877-883 of T7* RNAP. The P_{Tac} expression system and RBS are identical to those in the splitposon. (**D**) An example of a clone in the final bisection library. In this case, the splitposon is inserted in the forward direction into the T7* RNAP CDS. Plasmids pTHSSd_4-7, which were used to re-verify the 601 split and test the effect of adding SynZIPs (Fig 2E) look identical (plus the added SynZIPs). Both the expression plasmid and final library contain the p15A* origin and are spectinomycin resistant. Because of the splitposon, the final library is also kanamycin resistant.*

Supplementary Table S1. Statistics of T7 RNA polymerase bisection mapping.

Library	Library variants[a]	Harvested colonies[b]	Library coverage[c]
Transposon Insertion	9026	7.8 x10^5	87
Final	4564	6.0 x10^5	132

a. *The number of possible variants in the insertion and final split T7* RNAP libraries. Equal to 2x the size of the insertion plasmid and 2x the size of the T7* RNAP 41-876 fragment, respectively.*
b. *The approximate number of colonies scraped and pooled for the two libraries, determined by plating dilutions and counting colonies.*
c. *The harvested clones divided by the number of variants in each library.*

II.C. Split sites shown on the T7 RNAP structure

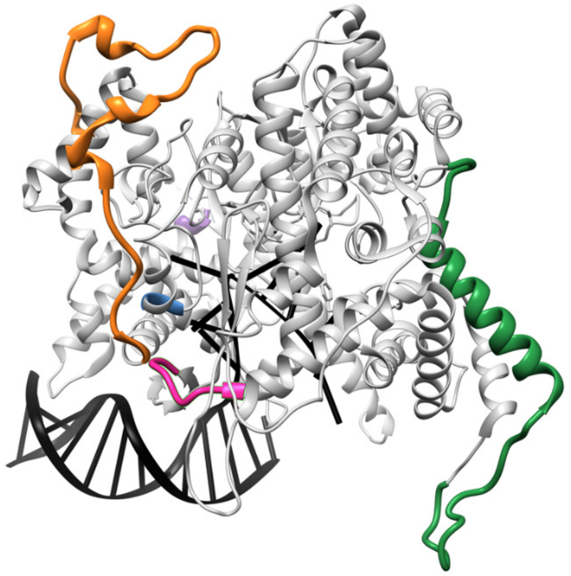

Supplementary Figure S3. *The five seams identified in Fig 2B are shown on the T7 RNAP transcribing initiation complex structure (*PDB# = 1QLN (Cheetham & Steitz, 1999), visualized using UCSF Chimera (Pettersen *et al*, 2004)) *using the same color scheme: Purple = 67-74, Orange = 160-206, Blue = 301-302, Green = 564-607, Pink = 763-770. DNA and the nascent RNA strand are shown in black.*

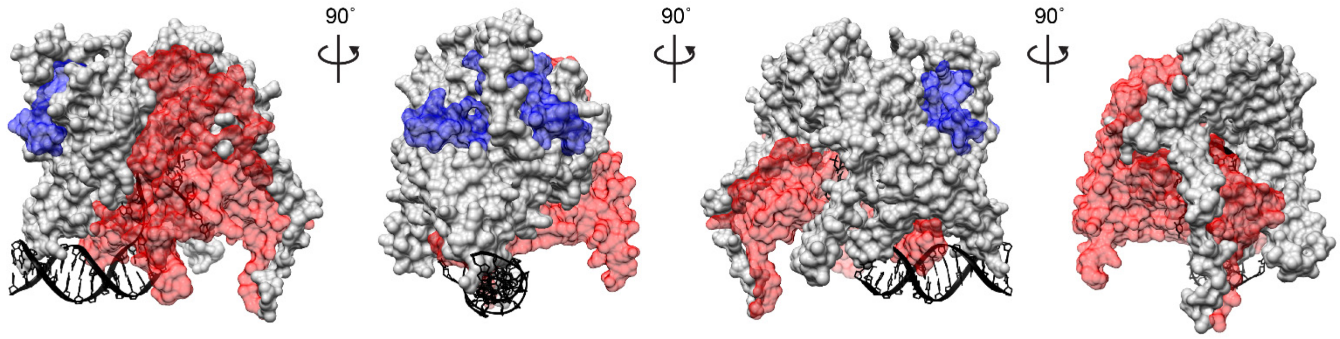

Supplementary Figure S4. Surface model of three-piece T7 RNAP. *A surface model of the T7 RNAP transcribing initiation complex structure (*PDB# = 1QLN, visualized using UCSF Chimera) *is shown, with the α fragment colored blue, the β core fragment colored grey, and the σ fragment colored red. The leftmost view shows transcription from left to right, and each subsequent image is rotated 90° around the y axis. DNA and the nascent RNA strand are shown in black.*

III. Supporting experiments

III.A. Directed evolution of the K1F and N4 σ fragments

Error-prone PCR was applied to increase the activity of σ fragments based on the K1F and N4 RNAP variants (Temme *et al*, 2012). After a visual screen for fluorescence, a number of clones with increased activity were identified for each σ fragment (Supplementary Tables S2 and S3). Nearly the full K1F σ fragment (residues 610-871 in the full-length polymerase) was mutated and screened for function. 13 highly active clones from this library were assayed and sequenced, revealing that 100% contained a point mutation affecting the residue corresponding to 750 in the full polymerase sequence. Based on these results, a variant of the K1F σ fragment was created with the M750R mutation (K1FR), which exhibits activity within 4-fold that of the T7 σ fragment and was used in all remaining experiments. The error-prone PCR protocol was applied to a smaller region of the N4 σ fragment (residues 716-789 in the full-length polymerase), and 12 improved clones were sequenced, but no sufficiently active mutations were found.

Supplementary Table S2. Improved activity clones from the K1F σ fragment ePCR library.

Clone #[a]	Mutations[b,c]		
1	**M750K**	Y746H	
2	K721R	**M750K**	
3	E694G	**M750K**	
4	**M750K**		
5	Q669R	**M750K**	
6	**M750K**		
7	Q744R	**M750V**	H772Y
8	**M750V**	H772R	E755K
9	**M750V**	H772R	E855K
10	**M750K**	Q786H	
11	E652K	**M750K**	
12	Q669R	**M750R**	
13	**M750R**	K826R	

a. *The clones are ordered from least to most active.*
b. *Residues are numbered by their position in the full-length T7 RNAP sequence.*
c. *Mutations affecting residue 750 are shown in bold.*

Supplementary Table S3. Improved activity clones from the N4 σ fragment ePCR library.

Clone #[a]	Mutations[b,c]	
1	H755R	
2	H755R	
3	H755R	
4	H755R	
5	H755R	
6	H755R	
7	H755R	
8	H755R	
9	H755R	
10	V725A	H772R
11	H755R	
12	H755R	

a. *The clones are ordered from least to most active.*
b. *Residues are numbered by their position in the full-length T7 RNAP sequence.*
c. *Additional silent mutations were found in #1 and #4.*

III.B. Means and error underlying the σ fragment orthogonally matrix

The data used to generate the orthogonality heatmap in Fig 3E are shown with error bars (Supplementary Fig S5). The promoter activity was measured for each σ fragment with each promoter, and each promoter in the absence of a σ fragment. Dividing the level of activity with each σ fragment by the level of activity without a σ fragment yields the fold induction. This data is also available in the source data file for Fig 3D-E.

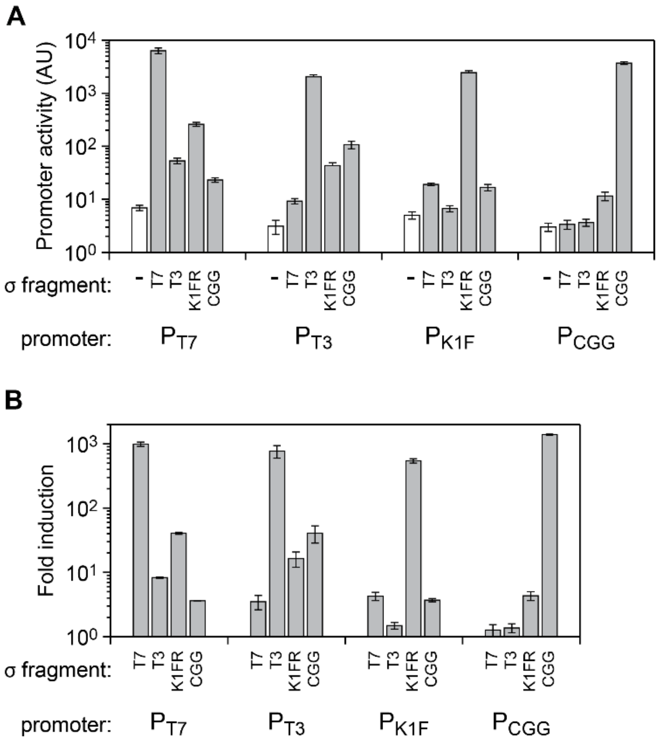

Supplementary Figure S5. Detailed σ fragment orthogonality results. (A) *Each of the σ fragments and a negative control were induced with 10 μM IPTG in the presence of the core fragment and each of the four promoters. Grey bars represent promoter activity with expressed σ fragments, white bars indicate the promoter activity of negative controls with no expressed σ fragment. (**B**) The fold induction of each σ fragment in combination with each promoter is shown. Each bar represents the mean value of three independent assays performed on different days, with error bars showing standard deviation.*

III.C. Identifying the null fragment

To determine the optimal null fragment, three known inactivating mutations (Bonner *et al*, 1992; Mookhtiar *et al*, 1991) were tested in the background of three σ fragments. The intention was to find a mutation that abolishes polymerase function without inhibiting the ability of the null fragment to compete with other σ fragments to bind the core fragment. A deletion of residues 882-3 and two point mutations, Y639A and H811A, were tested. These mutations were made to the T7 σ fragment, the CGG σ fragment, and a σ fragment based on WT T7 RNAP (rather than T7* RNAP as for the T7 σ fragment). The T7 σ fragment was expressed constitutively with the core fragment and a P_{T7} reporter plasmid, and the variant null fragments were induced with IPTG. By comparing the P_{T7} promoter activity with and without induction of the null fragments, a fold repression value was calculated for each variant (Supplementary Table S4). Based on this data, The CGG σ fragment with mutation Y639A was found to be the most active and was chosen as the null fragment. To determine whether the null fragment retains residual activity, it was expressed with the core fragment and a P_{CGG} reporter. Even at high levels of induction, this null fragment shows no P_{CGG} promoter activity when expressed with the core fragment (Supplementary Fig S6).

Supplementary Table S4. Comparison of null fragment variants.

Null variant	Fold repression[a]
σ_{T7} Δ882-3	9
σ_{T7} Y639A	12
σ_{T7} H811A	11
σ_{CGG} Δ882-3	14
σ_{CGG} Y639A	18
σ_{CGG} H811A	16
σ_{T7WT} Δ882-3	12
σ_{T7WT} Y639A	13
σ_{T7WT} H811A	13

a. *Fold repression was calculated as the activity of a P_{T7} promoter with constitutive σ_{T7} expression and no null fragment induction (0 μM IPTG) divided by the activity of the P_{T7} promoter with constitutive σ_{T7} expression and high null fragment induction (1000 μM IPTG). Values are the mean fold repression from three independent assays performed on different days.*

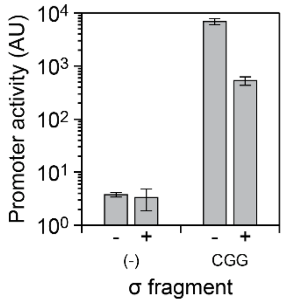

Supplementary Figure S6. The null fragment lacks σ fragment activity. *The null fragment is induced from P_{Tac} with 0 (-) or 1000 μM (+) IPTG in the presence of the core fragment, a P_{CGG} reporter and either the CGG σ fragment (CGG) or no σ fragment (-). The mean promoter activity from three independent assays is shown, with error bars showing standard deviation.*

III.D. Activation of the β core fragment with proteins fused to the α fragment

We tested fusions of the α fragment to GFP for their ability to complement the β core fragment. Similar to the RFP-α fusion assays in Fig 5G-H, the GFP- α fusions were induced from P_{Tac} in the presence of constitutively expressed β core fragment and σ_{T7}. A reporter plasmid that produces RFP from a P_{T7} promoter was used to measure polymerase activity (Supplementary Fig S7).

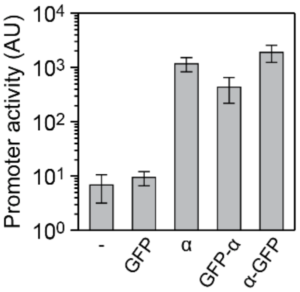

Supplementary Figure S7. Activity of GFP : α fragment fusions. *The ability of α : GFP fusions to complement constitutively expressed β core fragment and σ_{T7} is shown by the activity of a P_{T7} promoter driving RFP. (-): no inducible cassette, GFP: expression of unmodified GFP, α: expression of unmodified α fragment, GFP-α: expression of an N-terminal fusion of GFP to the α fragment, α-GFP: expression of a C-terminal fusion of GFP to the α fragment. Each system was induced with 40 μM IPTG. Each bar shows the mean level of activity from three independent assays, and error bars show the standard deviation.*

III.E. Measurement of P_{Tac} activity

In order to estimate the amount of RNAP fragments produced by our inducible plasmids, we measured GFP production from similar P_{Tac} expression plasmids (Supplementary Fig S9). The RiboJ insulator removes promoter context issues, leading to linear relationships between the expression levels of two proteins driven by identical promoters (Lou *et al*, 2012). Hence, the measured values for GFP production should linearly correlate with the RNAP fragments produced in each system.

P_{Tac} measurements were taken and plotted on the x-axis for the four the assays presented in Figs 3B, 4B,C,E, 5B, and 5E (Supplementary Fig S8). In each case, the P_{Tac} measurement was taken concurrently with the other measurements, from cells growing in the same conditions.

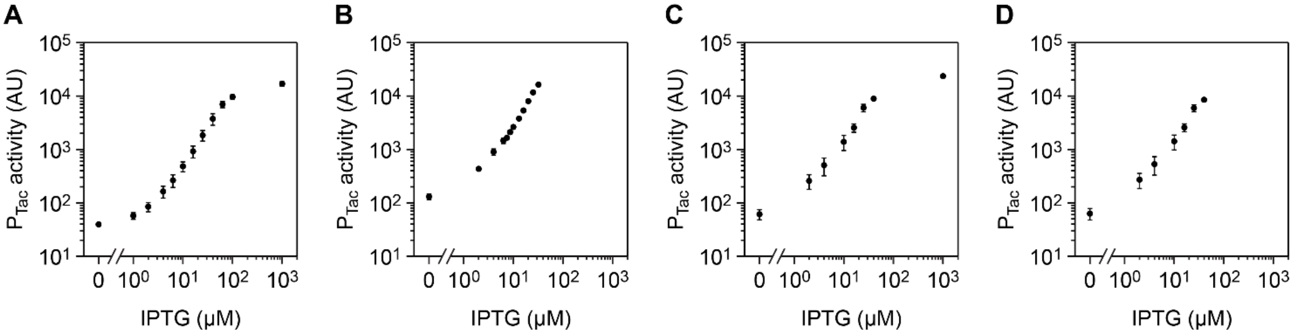

Supplementary Figure S8. ***P_{Tac} activity measurements.*** *Measurements of GFP production by P_{Tac} were taken under different conditions to determine relative expression levels in a number of assays. (**A**) P_{Tac} measurements for the assay in Fig 3B with plasmid pTHSSd_34. From left to right, expression was induced with 0, 1, 2, 4, 6.3, 10, 16, 25, 40, 63, 100, and 1000 μM IPTG. (**B**) P_{Tac} measurements for the assay in Fig 4B,C,E with plasmid pTHSSd_50. From left to right, expression was induced with 0, 2, 4, 6.3, 7.4, 8.6, 10, 13, 16, 20, 25, and 32 μM IPTG. (**C**) P_{Tac} measurements for the assay in Fig 5B with plasmid pTHSSd_34. From left to right, expression was induced with 0, 2, 4, 10, 16, 25, 40, and 1000 μM μM IPTG. (**D**) P_{Tac} measurements for the assay in Fig 5E with plasmid pTHSSd_34. From left to right, expression was induced with 0, 2, 4, 10, 16, 25, and 40 μM μM IPTG. For all graphs, the mean is shown for three independent assays performed on different days, with error bars showing standard deviation.*

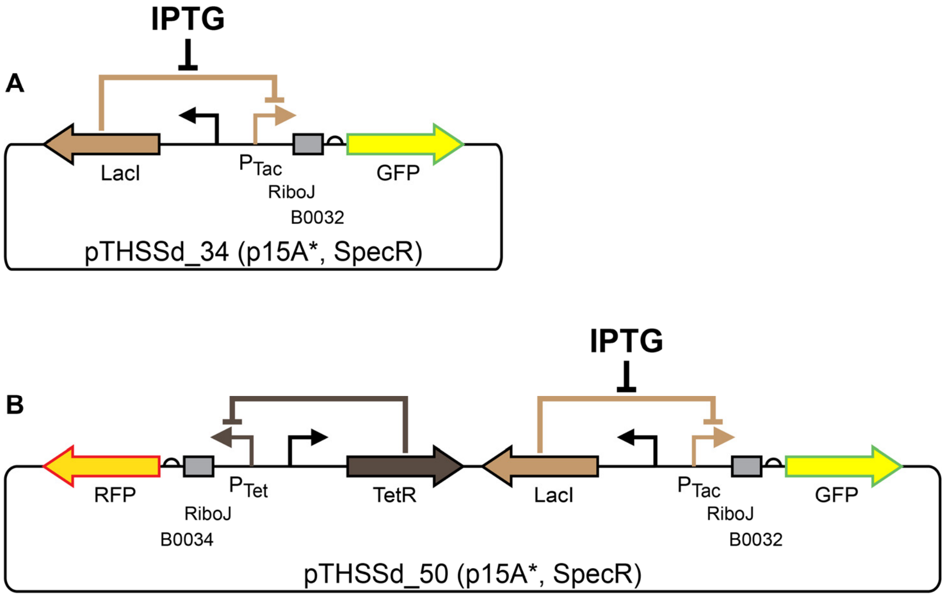

Supplementary Figure S9. Plasmids used for P_{Tac} activity measurements. (A) *pTHSSd_34 was used to characterize P_{Tac} expression in Figs 3B, 5B, and 5E. It expresses GFP under control of P_{Tac}, with RiboJ and the B0032 RBS. (**B**) pTHSSd_50 was used to characterize P_{Tac} expression in the σ fragment competition assay (Fig 4). It expresses GFP under the control of P_{Tac} with RiboJ and the B0032 RBS. Additionally, RFP is expressed under the control of P_{Tet}, with RiboJ and the B0034 RBS. Both plasmids have a p15A* origin with spectinomycin resistance.*

III.F. Growth impact of split polymerase expression

A number of the systems used to test split polymerase activity were measured to determine their impact on cell growth.T7 RNA polymerase is known to be toxic, especially when expressed in the presence of its promoter. Additionally, split proteins can be unstable and misfold, leading to further growth impacts.

We tested three systems of split polymerase expression for growth impacts: the four orthogonal σ fragment and core fragment expression systems shown in Fig 3A (Supplementary Fig S10), the σ fragment competition systems shown in Fig 4A (Supplementary Fig S11), and the multiply-split polymerase expression systems used in Figs 2D-E (Supplementary Fig S12). The full length T7* RNAP was also tested when expressed from the same system as is used for the multiply-split polymerases (Supplementary Figs S12, S13). Each of these expression systems was induced with varying levels of IPTG and compared to a negative control containing the appropriate plasmid backbones, but not expressing the polymerase fragments or fluorescent proteins.

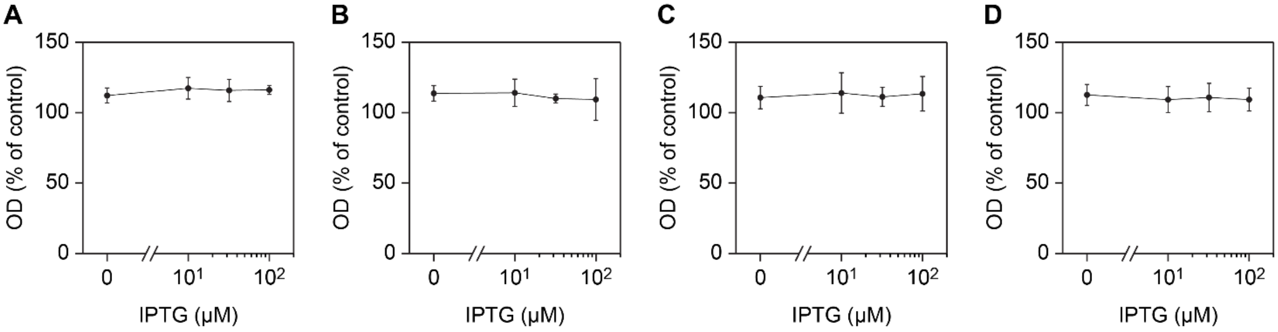

Supplementary Figure S10. Growth impact of orthogonal split polymerase systems. *The growth impact of the split polymerase expression systems from Figs 3B-C is shown. The four orthogonal σ fragments were expressed with IPTG (induction from left to right: 0, 10, 32, and 100 μM) in the presence of the core fragment (pTHSSd_38) with the appropriate reporter plasmid and the OD_{600} after 6 hours of growth compared to a control strain carrying plasmids that do not express the polymerase fragments or GFP (pTHSSd_36, pTHSSd_43, pTHSSd_13). (**A**) T7 σ fragment and reporter (pTHSSd_23 and pTHSSd_8). (**B**) T3 σ fragment and reporter (pTHSSd_24 and pTHSSd_9). (**C**) K1FR σ fragment and reporter (pTHSSd_25 and pTHSSd_10). **(D)** CGG σ fragment and reporter (pTHSSd_26 and pTHSSd_11). For all graphs, the mean is shown for three independent assays performed on different days, with error bars showing standard deviation.*

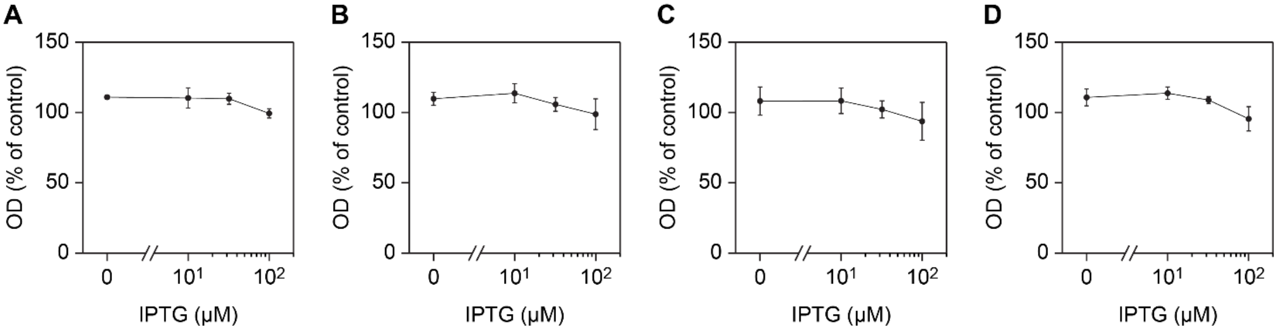

Supplementary Figure S11. Growth impact of σ fragment competition systems. *The growth impact of the competition systems from Fig 4 is shown. The T3 σ fragment was expressed with IPTG (induction from left to right: 0, 10, 32, and 100 μM) in the presence of the K1FR σ fragment (pTHSSd_49) and high or low levels of the core fragment with either the T3 or K1FR reporter plasmid and the OD_{600} after 6 hours of growth compared to a control strain carrying plasmids that do not express the polymerase fragments or GFP (pTHSSd_36, pTHSSd_43, pTHSSd_13). (**A**) Higher level of core fragment expression with the T3 reporter (pTHSSd_38, pTHSSd_9). (**B**) Higher level of core fragment expression with the K1FR reporter (pTHSSd_38, pTHSSd_10). (**C**) Lower level of core fragment expression with the T3 reporter (pTHSSd_39, pTHSSd_9). (**D**) Lower level of core fragment expression with the K1FR reporter (pTHSSd_39, pTHSSd_10). For all graphs, the mean is shown for three independent assays performed on different days, with error bars showing standard deviation.*

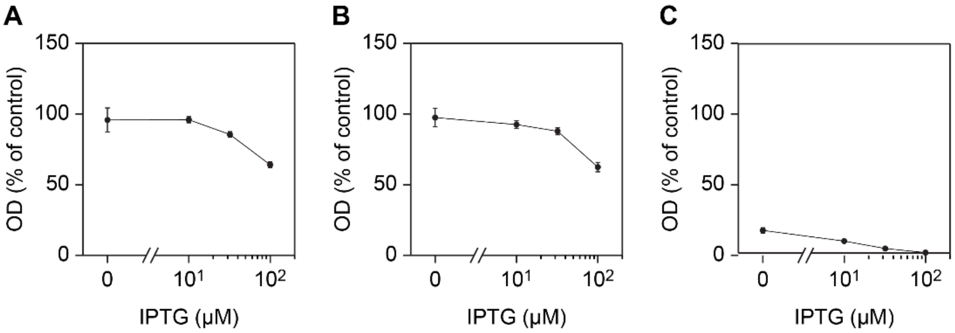

Supplementary Figure S12. Growth impact of highly expressed multiply split polymerase. *The growth impact of the three and four fragment polymerases from Figs 2D-E is shown with a full-length polymerase control. The split or full T7* polymerases were expressed with IPTG (induction from left to right: 0, 10, 32, and 100 μM) in the presence of a T7 reporter plasmid (pTHSSd_8) and the OD_{600} after 6 hours of growth compared to a control strain carrying plasmids that do not express the polymerase fragments or GFP (pTHSSd_36, pTHSSd_13). (**A**) Three piece T7* RNA polymerase (pTHSSd_14). (**B**) Four piece T7* RNA polymerase (pTHSSd_18). (**C**) Full-length T7* RNA polymerase (pTHSSd_37). For all graphs, the mean is shown for three independent assays performed on different days, with error bars showing standard deviation.*

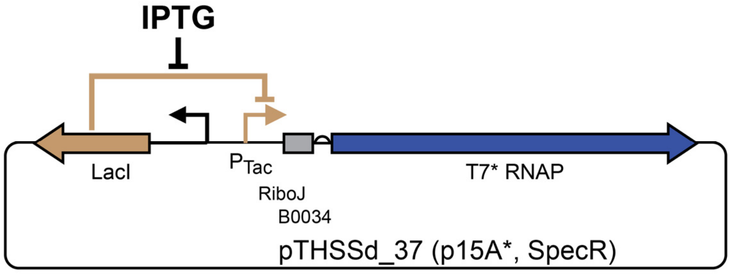

Supplementary Figure S13. Plasmid used for full-length T7* RNAP toxicity measurement. *pTHSSd_37 was used to characterize the growth impact of expressing T7* RNAP from the same expression system used to drive the thee- and four-piece polymerases. It contains the full T7* RNAP CDS driven by a* P_{Tac} *expression system with RiboJ and the B0034 RBS.*

IV. Mathematical models

IV.A. Kinetic model of the resource allocator

We used a kinetic model to examine the contrasting outcomes on total active RNAPs in a system with or without the resource allocator (Figs 1B-C). In each case, four promoters on the controller are modeled driving either four RNAPs or σ fragments. The promoters are switched between fully off and fully on states at different time points.

In the model with expression of full-length RNAPs (Fig 1B), only two reactions per RNAP were considered, yielding one equation per RNAP:

$$\dot{r}_i = u_i - \gamma r_i \qquad i = 1 - 4 \tag{1}$$

where the dot indicates a time derivative, and:

- $r_i = r_i(t) \geq 0$ is the concentration of the ith full-length RNAP,
- u_i is the lumped transcription and translation rate of the ith RNAP, and
- γ is the degradation rate (assumed equal) of the RNAPs.

For the model involving the resource allocator (Fig 1C), a number of additions were made. A core polymerase fragment is produced at a fixed rate equal to RNAP production in the previous model, while the four promoters now drive σ fragments of the polymerase. The σ fragments can bind the core fragment to form full-length RNAP complexes which can dissociate back into σ and core fragments. Again, all degradation rates are assumed to be equal. This yields the following three equations:

$$\dot{\sigma}_i = u_i - \gamma\sigma_i + k_d r_i - k_a\sigma_i c \qquad i = 1 - 4 \tag{2}$$

$$\dot{r}_i = -\gamma r_i - k_d r_i + k_a\sigma_i c \qquad i = 1 - 4 \tag{3}$$

$$\dot{c} = v - \gamma c + k_d(\textstyle\sum_{i=1}^{k} r_i) - k_a(\textstyle\sum_{i=1}^{k}\sigma_i c) \tag{4}$$

where dots indicate time derivatives, and:

- $\sigma_i = \sigma_i(t) \geq 0$ is the concentration of (unbound) σ fragment i,
- $r_i = r_i(t) \geq 0$ is the concentration of the ith full-length RNAP complex,
- $c = c(t) \geq 0$ is the concentration of core fragment,
- u_i, v are the lumped transcription and translation rates of the ith σ fragment and the core fragment, respectively,
- γ is the degradation rate (assumed equal) of the σ fragments, full-length RNAP complexes, and the core fragment,
- k_a is association rate of the σ fragments and the core fragment (assumed equal), and
- k_d is the dissociation rate of full-length RNAP's into σ fragments and the core fragment (again assumed equal)

We simulated time courses of RNAP concentration using these systems of equations and a set of estimated parameters (Supplementary Table S5). The degradation rate, γ , was assumed to be dominated by dilution through cell growth and equal for all species in the system. The lumped transcription and translation rate of the full-length RNAPs was set to yield a steady-state concentration of 0.1 μM when they are expressed, the rate for the core fragment was set to be the same, and the rate for the σ fragments was set to yield 0.2 μM when expressed. Finally, the rates for the σ fragments binding and unbinding the core fragment were based on an *in vitro* analysis of a heterodimeric coiled-coil interaction (Chao *et al*, 1996). Simulations were performed in MATLAB using the ode45 solver.

Supplementary Table S5. Modeling parameters

Parameter	RNAP model	Resource allocator model
γ	$3x10^{-4}s^{-1}$	$3x10^{-4}s^{-1}$
$u_i(off)$	$0Ms^{-1}$	$0Ms^{-1}$
$u_i(on)$	$3x10^{-11}Ms^{-1}$	$6x10^{-11}Ms^{-1}$
v	-	$3x10^{-11}Ms^{-1}$
k_a	-	$4.5x10^{5}Ms^{-1}$
k_d	-	$2x10^{-4}s^{-1}$

IV.B. Uniqueness and stability of steady states in resource allocator model

We study the resource allocator model in (2-4), with the following changes:

- There are k different σ fragments and RNAPs rather than limiting to 4: $i = 1, \ldots, k$
- The lumped transcription and translation rates, u_i and v, are assumed constant and positive.

For simplicity, we also write the system in vector form as

$$\dot{x} = f(x) \qquad (S)$$

where

$$x(t) = (\sigma_1(t), \ldots, \sigma_k(t), r_1(t), \ldots, r_k(t), c(t)) .$$

In general, a system of nonlinear ODE's (S) might have multiple stable states or persistent oscillations, or even exhibit chaotic behavior. It is thus of interest to show mathematically that our model has none of these, and, as a matter of fact, has the property that all solutions converge to a unique steady state, independently of initial concentrations. This is proved in the following result.

Theorem. There is a unique non-negative steady state of (S), which we will denote as

$$\bar{x} = (\bar{\sigma}_1, \ldots, \bar{\sigma}_k, \bar{r}_1, \ldots, \bar{r}_k, \bar{c}) .$$

Moreover, every solution of (S) with $x(t) \geq 0$ satisfies $x(t) \to \bar{x}$ as $t \to \infty$.

Proof. It is convenient to introduce, for any given solution $x(t)$, the following combinations of variables:

$$s_i(t) := \sigma_i(t) + r_i(t) , \quad i = 1, \ldots, k \quad \text{(total } \sigma \text{ fragment } i\text{, bound and unbound)}$$

$$\sigma(t) := \sum_{i=1}^{k} \sigma_i(t) , \quad i = 1, \ldots, k \quad \text{(total unbound } \sigma \text{ fragments)}$$

$$s(t) := \sum_{i=1}^{k} s_i(t) \quad \text{(total } \sigma \text{ fragments, bound and unbound).}$$

$$r(t) := \sum_{i=1}^{k} r_i(t) \quad \text{(total RNAP complexes, without unbound core fragments).}$$

Observe that

$$\sigma(t) = s(t) - r(t)$$

for all t, or equivalently $s(t) = \sigma(t) + r(t)$. Since $\sigma(t) \geq 0$, it holds that

$$r(t) \leq s(t) \qquad (5)$$

for all t. We also introduce

$$R(t) := c(t) + r(t) \quad \text{(total core fragments, bound and unbound).}$$

Since $c(t) \geq 0$, it holds that

$$r(t) \leq R(t) \qquad (6)$$

for all t.

For each $i \in \{1, \ldots, k\}$, we have:

$$\dot{s}_i \;=\; \dot{\sigma}_i + \dot{r}_i \;=\; u_i - \gamma\sigma_i - \gamma r_i \;=\; u_i - \gamma s_i \,.$$

Therefore, along any solution,

$$\lim_{t\to\infty} s_i(t) \;=\; \bar{s}_i \;:=\; \frac{u_i}{\gamma} \tag{7}$$

and so also

$$\lim_{t\to\infty} s(t) \;=\; \bar{s} \;:=\; \frac{1}{\gamma}\sum_{i=1}^{k} u_i \,. \tag{8}$$

Similarly, for R we have:

$$\dot{R} \;=\; \dot{c} + \sum_{i=1}^{k} \dot{r}_i \;=\; v - \gamma c - \gamma(\textstyle\sum_{i=1}^{k} r_i) \;=\; v - \gamma R$$

and therefore, along any solution,

$$\lim_{t\to\infty} R(t) \;=\; \bar{R} \;:=\; \frac{v}{\gamma} \,. \tag{9}$$

Consider now an arbitrary steady state $\hat{x} = (\hat{\sigma}_1, \ldots, \hat{\sigma}_k, \hat{r}_1, \ldots, \hat{r}_k, \hat{c})$. Let $\hat{s}_i := \hat{\sigma}_i + \hat{r}_i$ ($i = 1, \ldots, k$), $\hat{\sigma} := \sum_{i=1}^{k} \hat{\sigma}_i$, $\hat{s} := \sum_{i=1}^{k} \hat{s}_i$, $\hat{r} := \sum_{i=1}^{k} \hat{r}_i$, and $\hat{R} := \hat{c} + \hat{r}$. Because of the above remarks, it must hold that $\hat{s}_i = \bar{s}_i$ ($i = 1, \ldots, k$), $\hat{s} = \bar{s}$, and $\hat{R} = \bar{R}$.

Along any trajectory, r satisfies the following differential equation:

$$\dot{r} \;=\; -(\gamma + k_d)r + k_a\sigma c \;=\; -(\gamma + k_d)r + k_a(s - r)(R - r) \,. \tag{10}$$

Note that this is a quadratic differential equation with time-dependent coefficients (since R and s are time-dependent functions). We study its stability behavior below, but first note that, at any steady state, since $R = \bar{R}$ and $s = \bar{s}$, the steady state value $\hat{r}$ must satisfy:

$$(\gamma + k_d)\hat{r} \;=\; k_a(\bar{s} - \hat{r})(\bar{R} - \hat{r}) \,. \tag{11}$$

It is convenient to introducing the following constant, which can be thought of as an effective dissociation constant for RNAP complexes:

$$K \;=\; \frac{\gamma + k_d}{k_a} \,,$$

we can rewrite (11) as

$$K\hat{r} \;=\; (\bar{s} - \hat{r})(\bar{R} - \hat{r}) \,. \tag{12}$$

As a function of $\hat{r}$, the left-hand side of (11) is a linear function with positive slope which vanishes at zero, and the right-hand side is a parabola opening up, with roots at $\bar{R}$ and $\bar{s}$. Thus, there is exactly one solution of (11), which we call $\bar{r}$, that is less than max$\{\bar{R}, \bar{s}\}$, and, in fact, is less than min$\{\bar{R}, \bar{s}\}$. An explicit formula for $\bar{r}$ (not required for the proof) is:

$$\bar{r} \;=\; \frac{1}{2k_a}(B - \sqrt{D}) \quad \text{where} \quad B = \gamma + k_d + k_a\bar{R} + k_a\bar{s}\,,\; D = B^2 - 4k_a^2\bar{R}\bar{s}\,.$$

By (5) and (6), $r(t) \leq s(t)$ and $r(t) \leq R(t)$ along all solutions (including constant solutions), so certainly $\hat{r} \leq$ min$\{\bar{R}, \bar{s}\}$, and thus $\hat{r} = \bar{r}$. Therefore

$$\hat{c} = \hat{R} - \hat{r} = \bar{c} := \bar{R} - \bar{r} \,. \tag{13}$$

Using that $\sigma_i = s_i - r_i$, we have, for each r_i:

$$\dot{r}_i \;=\; -(\gamma + k_d) r_i + k_a (s_i - r_i) c \;=\; k_a s_i c - (\gamma + k_d + k_a c) r_i \,. \tag{14}$$

So, at any steady state, since $s_i = \bar{s}_i$ and $c = \bar{c}$:

$$\hat{r}_i \;=\; \bar{r}_i \;:=\; \frac{k_a \bar{s}_i \bar{c}}{\gamma + k_d + k_a \bar{c}} \;=\; \frac{\bar{s}_i \bar{c}}{K + \bar{c}}\,, \tag{15}$$

which is formally analogous to a Michaelis-Menten product formation law. Notice that, as a consequence of (15), $\hat{r}_i/\hat{r}_j = \bar{s}_i/\bar{s}_j$ for any two i, j, and, in view of (7),

$$\frac{\hat{r}_i}{\hat{r}_j} \;=\; \frac{u_i}{u_j} \tag{16}$$

for all $i, j \in \{1, \dots, k\}$, which means that the RNAP complexes are produced in the same proportion as the proportion between the respective inputs. It also follows that

$$\hat{\sigma}_i = \hat{s}_i - \hat{r}_i = \bar{\sigma}_i := \bar{s}_i - \bar{r}_i \,. \tag{17}$$

Defining $\bar{x}$ by the formulas in (17), (15), (13), we conclude that $\hat{x} = \bar{x}$, and the steady state is indeed unique.

Next, we show that $x(t) \to \bar{x}$ as $t \to \infty$, for every solution. If we assume that $s(t)$ and $R(t)$ are already at their steady states given by (8) and (9), the differential equation (10) becomes:

$$\dot{r} \;=\; -(\gamma + k_d) r + k_a (\bar{s} - r)(\bar{R} - r)\,. \tag{18}$$

(A justification for the assumption that R and s can be assumed to be at steady state will be given later.) The right-hand side of this ODE is the difference between the two sides in (11), and thus is positive on $0 \le r < \bar{r}$ and negative on $\bar{r} < r \le \bar{R}$. Recall that we are only interested in solutions for which $r(t) \le \bar{R}$. Therefore $r(t) \to \bar{r}$ as $t \to \infty$. Since $c(t) = R(t) - r(t)$, it follows that from the definition $\bar{c} = \bar{R} - \bar{r}$ that

$$\lim_{t \to \infty} c(t) \;=\; \bar{c}\,. \tag{19}$$

If we assume (justified later) that $s(t)$ and $c(t)$ are already at their steady states given by (8) and (19), the differential equation (14) becomes:

$$\dot{r}_i \;=\; k_a \bar{s}_i \bar{c} - (\gamma + k_d + k_a \bar{c}) r_i \,. \tag{20}$$

for each $i = 1, \dots, k$. This is a stable linear constant-coefficient differential equation, so

$$\lim_{t \to \infty} r_i(t) \;=\; \bar{r}_i \tag{21}$$

for every i. Finally, from $\sigma_i(t) = s_i(t) - r_i(t)$, the definition $\bar{\sigma}_i = \bar{s}_i - \bar{r}_i$, together with (7) and (21), we have that

$$\lim_{t \to \infty} \sigma_i(t) \;=\; \bar{\sigma}_i \tag{22}$$

for every i. We have thus proved that $x(t) \to \bar{x}$ as $t \to \infty$.

Since (16) says that $\bar{r}_i/\bar{r}_j = u_i/u_j$ for all $i, j \in \{1, \dots, k\}$, we have then that, for any arbitrary $j \in \{1, \dots, k\}$:

$$\bar{r} \;=\; \sum_{i=1}^{k} \bar{r}_i \;=\; \sum_{i=1}^{k} \frac{u_i}{u_j} \bar{r}_j \;=\; \frac{\sum_{i=1}^{k} u_i}{u_j} \bar{r}_j$$

or equivalently:

$$\bar{r}_j \;=\; \bar{r}\left(\frac{u_j}{\sum_{i=1}^k u_i}\right) \tag{23}$$

which means that the relative expression of the jth RNAP complex is directly proportional to the fraction of its respective control input. For example, suppose that $k = 2$, and u_1 is maintained constant. Then the expression of the second RNAP complex at steady state has the hyperbolic Michaelis-Menten form $\bar{r}_2 = \frac{Vu_2}{u_1+u_2}$, where $V = \bar{r}$.

Justification of quasi-steady state assumption

It only remains to justify the hypotheses made at two points that variables already shown to approach steady state can be replaced by their steady state values in other equations (this is sometimes called the ``CICS'' or ``convergent input to convergent state property''). One way to prove this is to appeal to the theory of asymptotically autonomous systems: we view (10) as a non-autonomous differential equation which, as $t \to \infty$, approaches the autonomous equation (18). Since this latter equation has $\bar{r}$ as a globally asymptotically stable state (for initial conditions in, for example, the interval $[0, \max\{\bar{R}, \bar{s}\}]$), it follows that solutions of (18) also approach $\bar{r}$. (See the last section in (Ryan & Sontag, 2006) for details of this technique and further references.) Similar considerations apply to the linear ODE (14) and its limit equation (20).

Simplifications when $K \ll 1$

For realistic degradation and association and dissociation constants, K is very small, typically $\approx 10^{-9}$M. In that case, the formulas for steady state values can be simplified considerably. We will assume that $v < \sum_{i=1}^k u_i$ (the core fragment is the limiting factor), in which case $\bar{R} = v/\gamma < (\sum_{i=1}^k u_i)/\gamma = \bar{s}$, and thus $\min\{\bar{R}, \bar{s}\} = \bar{R}$. When $K \approx 0$, the unique steady state value $\bar{r} \leq \bar{R}$ that solves $(\bar{s} - \bar{r})(\bar{R} - \bar{r}) = K\bar{r} \approx 0$ is $\bar{r} \approx \bar{R}$. This means that (23) is, more explictly:

$$\bar{r}_j \;\approx\; \bar{R}\left(\frac{u_j}{\sum_{i=1}^k u_i}\right) \;=\; \frac{v}{\gamma}\left(\frac{u_j}{\sum_{i=1}^k u_i}\right) \tag{24}$$

It is important to note, however, that informal approximation arguments are not mathematically rigorous, and can easily lead to paradoxical conclusions. For example, (13) implies that $\bar{c} = \bar{R} - \bar{r} \approx 0$ (since we had $\bar{R} \approx \bar{r}$), and this, combined with (15) gives that $\bar{r}_i = \frac{\bar{s}_i\bar{c}}{K+\bar{c}} \approx \frac{\bar{s}_i \times 0}{K+0} = 0$! (The fallacy in this case comes from the approximation ``$x/(K + x) \approx 0$ when $x \approx 0$'' which is false if $K \ll x$.)

To make the argument mathematically precise, let us think of the unique steady state value $\bar{r} \leq \bar{R}$ that solves $K\bar{r} \;=\; (\bar{s} - \bar{r})(\bar{R} - \bar{r})$ as a function $\bar{r}(K)$, and take its limit as $K \to 0$ while keeping $\bar{R}$ and the $\bar{s}_i$'s fixed. Keeping these values fixed is valid for example if $k_a \to \infty$, or if $k_d \to 0$ and $\gamma \to 0$ at the same time that the control inputs (v and the u_i's) are proportionally increased. Using implicit differentiation, and primes to indicate derivative with respect to K, we have that $\bar{r} + K\bar{r}' = -\bar{r}'(\bar{R} - \bar{r}) - \bar{r}'(\bar{s} - \bar{r})$. Since $\bar{r} = \bar{R}$ when $K = 0$, the derivative at $K = 0$ is $\bar{r}' = \bar{R}/(\bar{R} - \bar{s})$ and thus we obtain the first-order Taylor expansion

$$\bar{r}(K) \;=\; \bar{r}(0) + \bar{r}'(0)K \;+\; o(K) \;=\; \bar{R} + \frac{\bar{R}}{\bar{R} - \bar{s}}K \;+\; o(K)\,.$$

Then, $\bar{c} = \bar{R} - \bar{r} = \frac{\bar{R}}{\bar{s} - \bar{R}}K + o(K)$, and now substituting into $\bar{r}_j = \frac{\bar{s}_j\bar{c}}{K+\bar{c}}$, we conclude that:

$$\bar{r}_j \;=\; \frac{\bar{s}_j\bar{c}}{K + \bar{c}} \;=\; \bar{s}_j\frac{\bar{R}}{\bar{s}} + O(K) \;=\; \frac{v}{\gamma}\left(\frac{u_j}{\sum_{i=1}^k u_i}\right) + O(K)\,,$$

which recovers (24) as $K \to 0$.

IV.C. Modeling σ fragment competition data

Using the simplified steady-state equations presented in (24), we can model the σ fragment competition data shown in Fig 4. In the context of the experiments shown in Fig 4, there are only two σ fragments, T3 and K1FR, yielding the equations:

$$\bar{r}_{T3} \;\approx\; \bar{R}\left(\frac{u_{T3}}{u_{T3}+u_{K1F}}\right) \tag{25}$$

$$\bar{r}_{K1F} \;\approx\; \bar{R}\left(\frac{u_{K1F}}{u_{T3}+u_{K1F}}\right) \tag{26}$$

If the P_{T3} and P_{K1FR} promoter activities are linearly proportional to the concentration of the appropriate RNAP complex, these equations immediately predict the result shown in Fig 4D; changing the resource allocator results in an identical linear scaling of the promoter outputs. Changing the expression of the core fragment from the resource allocator changes the value of $\bar{R}$, which linearly scales $\bar{r}_{T3}$ and $\bar{r}_{K1FR}$ identically for any constant values of u_{T3} and u_{K1FR}.

In Fig 4E, we normalize the promoter activities of P_{T3} and P_{K1FR} by the maximum promoter activities obtained when the appropriate σ fragments are expressed to saturate the core fragment. Assuming that the promoter activities are linearly proportional to the amount of corresponding RNAP present in the system, these normalized values represent the fraction of the core fragment bound by each σ fragment. That is $\bar{r}_{T3}/\bar{R}$, $\bar{r}_{K1F}/\bar{R}$, for the normalized activity values of P_{T3} and P_{K1FR}, respectively. Therefore, we have:

$$N_{T3} \;=\; \bar{r}_{T3}/\bar{R} \;\approx\; \left(\frac{u_{T3}}{u_{T3}+u_{K1F}}\right) \tag{27}$$

$$N_{K1F} \;=\; \bar{r}_{K1F}/\bar{R} \;\approx\; \left(\frac{u_{K1F}}{u_{T3}+u_{K1F}}\right) \tag{28}$$

where N_{T3} and N_{K1F} are the normalized P_{T3} and P_{K1FR} promoter activities shown in Fig 4E.

Finally, we have a relative measurement for the expression of the T3 σ fragment: the P_{Tac} expression level with the appropriate amount of inducer. Assuming that this value is linearly proportional to the true expression level of the T3 σ fragment, we can say: $u_{T3} = cP_{Tac}$, where c is a scaling factor to relate the P_{Tac} expression level to the σ_{T3} expression level. Substituting this into the model yields:

$$N_{T3} \;\approx\; \left(\frac{P_{Tac}}{P_{Tac}+\frac{u_{K1F}}{c}}\right) \tag{29}$$

$$N_{K1F} \;\approx\; \left(\frac{\frac{u_{K1F}}{c}}{P_{Tac}+\frac{u_{K1F}}{c}}\right) \tag{30}$$

As the N_{T3}, N_{K1F}, and P_{Tac} values are all measured, there is only one remaining free variable: $\frac{u_{K1F}}{c}$, which represents the constant expression level of the K1FR σ fragment in the same units as the P_{Tac} expression value. This parameter was determined by simultaneously fitting (29) and (30) to the N_{T3} and N_{K1F} data shown in Fig 4E, using a least-squares algorithm (lsqnonlin) in MATLAB. This yields a value of 617 for $\frac{u_{K1F}}{c}$. Hence, the final models shown in Fig 4E are:

$$N_{T3} \;\approx\; \left(\frac{P_{Tac}}{P_{Tac}+617}\right) \tag{31}$$

$$N_{K1F} \;\approx\; \left(\frac{617}{P_{Tac}+617}\right) \tag{32}$$

And the sum of those two equations:

$$N_{Sum} = N_{T3} + N_{K1F} \;\approx\; 1 \tag{33}$$

V. Plasmid details

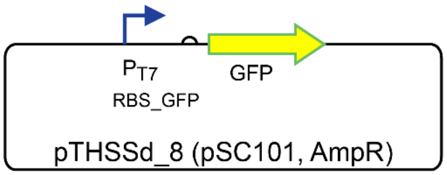

Supplementary Figure S14. ***Reporter plasmids.*** *The reporter constructs used in this work are based on plasmid pUA66* (Zaslaver *et al*, 2006)*, which has a pSC101 origin of replication. The GFPmut2 gene is replaced with sfGFP* (Pédelacq *et al*, 2006)*, and the kanamycin resistance cassette is replaced with an ampicillin resistance cassette. Variants were created with the* P_{T7}*,* P_{T3}*,* P_{K1F}*, and* P_{CGG} *promoters driving expression of GFP (pTHSSd_8-11). A strong RBS (RBS_GFP: TGTCAATTTCCGCGATAGAGGAGGTAAAG) was generated using the RBS calculator and used to control translation of GFP. For assaying GFP : α fragment fusions, a reporter variant was built with the* P_{T7} *mRFP1 expression cassette from NiF_489* (Temme *et al*, 2012) *(pTHSSd_12). A negative control plasmid lacking the GFP expression cassette was also generated (pTHSSd_13).*

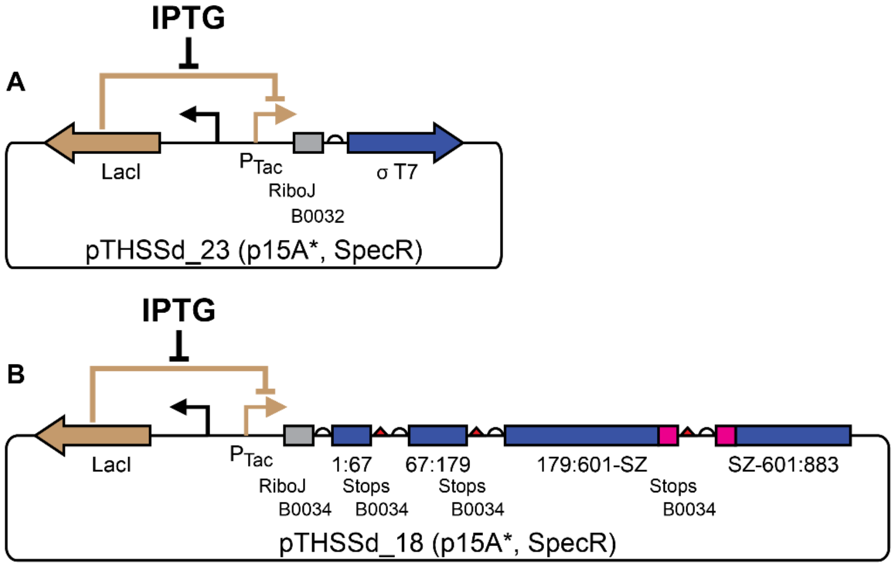

Supplementary Figure S15. ***Inducible expression plasmids.*** *Plasmids for the inducible expression of genes from* P_{Tac} *are built from pSB3C5* (Shetty *et al*, 2008)*, which has a p15A origin. This origin appears to maintain at a higher copy number than standard, so we refer to it as p15A*. The chloramphenicol resistance cassette is replaced with a spectinomycin resistance cassette, and a modified section from pEXT20* (Dykxhoorn *et al*, 1996) *containing a LacI expression cassette, a random spacer, and short* P_{Tac} *promoter is inserted into the plasmid. The lacO binding site in* P_{Tac} *is mutated to be symmetric (AATTGTGAGCGCTCACAATT), and is followed by RiboJ* (Lou et *al*, 2012). ***(A)*** *In most systems, only one coding sequence is expressed under the control of* P_{Tac} *and the B0032 RBS (BBa_B0032) is used. A number of proteins were expressed from plasmids similar to this, including σ fragments (pTHSSd_23-26), the null fragment (pTHSSd_27), the α fragment (pTHSSd_29), α : FP fusions (pTHSSd_30-33), and an RFP only control for the α : FP fusion test (pTHSSd_28).* ***(B)*** *To test T7* RNAP fragmented into three or four fragments, plasmids were constructed that express the fragments or a subset of them on one cistron (pTHSSd_14-22). The B0034 RBS (BBa_B0034) is used for each fragment, and a double stop codon terminates each fragment coding sequence. Two negative control plasmids were made that lack any inducible gene but contain LacI (pTHSSd_35, 36). pTHSSd_35 contains the LacI cassette and* P_{Tac} *promoter system found in the splitposon and bisection library, while pTHSSd_36 only contains the LacI expression cassette.*

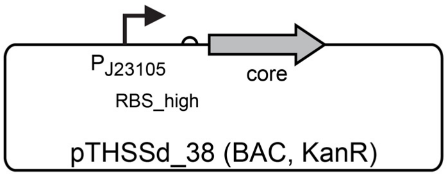

Supplementary Figure S16. ***Core fragment expression plasmids.*** *The core and β core fragments are expressed from plasmids based on pBACr-Mgr940* (Anderson *et al*, 2007) *(BBa_J61039), which carries kanamycin resistance and an F plasmid derived origin. The constitutive P_{J23105} promoter (BBa_J23105) is used to drive expression of the core fragment, core fragment variants, the full T7* RNAP, or β core fragment (pTHSSd_38-42), using different ribosome binding sites to control the strength of expression. The main RBSs used were derived from a degenerate library based on B0032: RBS_high (TACTAGAGTCATTTATGAAAGTACTAG) is used for most constructs, RBS_low: (TACTAGAGTCAGCCAAGAAAGTACTAG) is used for the lower level of core fragment expression. B0032 is used in the β core expression plasmid. A negative control of this plasmid was constructed that lacks an RBS and coding sequence (pTHSSd_43).*

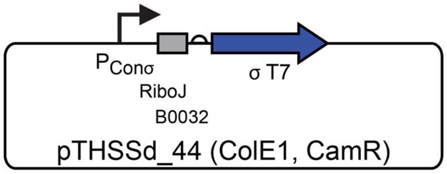

Supplementary Figure S17. ***Constitutive σ fragment expression plasmids.*** *For the null fragment and α fragment assays, σ fragments were constitutively expressed from plasmids based on pSB1C3* (Shetty *et al*, 2008)*, which has a ColE1 origin and chloramphenicol resistance. A variant of the constitutive promoter P_{J23105} ($P_{Con\sigma}$: TTGACAGCTAGCTCAGTCCTAGGCTATAGGCTAG), RiboJ, and the B0032 RBS are used to drive expression of each of the four σ fragments (pTHSSd_44-47). A negative control was made that lacks any piece of the expression cassette (pTHSSd_48).*

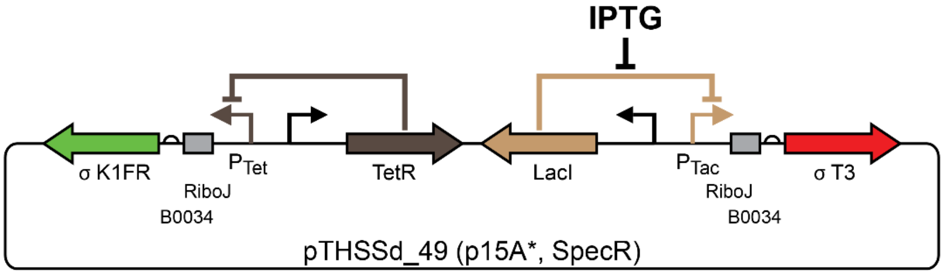

Supplementary Figure S18. ***σ fragment competition plasmids.*** *A variant of the T3 σ fragment inducible expression plasmid was built to test σ fragment competition. A modified P_{Tet} expression system* (Moon *et al*, 2012) *is added behind the P_{Tac} expression system facing in the reverse direction. The P_{Tet} promoter is followed by RiboJ and drives expression of the K1FR σ fragment. Both σ_{T3} and σ_{K1FR} use the B0034 RBS.*

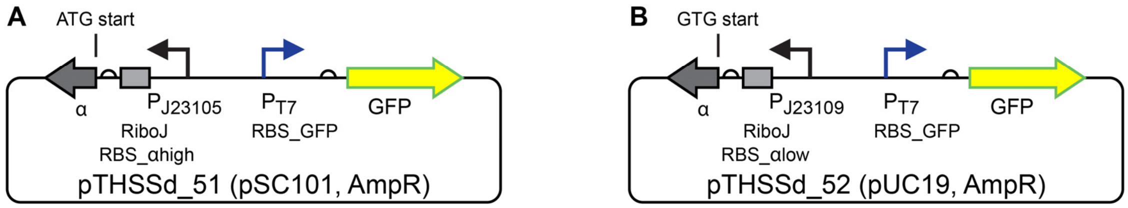

Supplementary Figure S19. Reporter plasmids with α fragment compensation. (A) *A constitutive α fragment expression cassette is added in the reverse direction to the P_{T7} reporter plasmid before the P_{T7} promoter to make pTHSSd_51. This cassette drives production of the α fragment with P_{J23105}, RiboJ, and a RBS derived from B0032 (RBS_αhigh: TCAACCACGAAAGTACTAG). (**B)** pTHSSd_52 has the same two cassettes as pTHSSd_51, inserted into a pUC19* (Yanisch-Perron *et al*, 1985, 19) *ampicillin resistant backbone. The α fragment cassette is changed to lower its expression level: the promoter is switched to P_{J23109} (BBa_J23109), a different RBS is used (RBS_αlow: CTAGTACTTTCGTTCATGA), and the α fragment start codon is changed to a GTG from ATG.*

Supplementary Table S6. New plasmids used in this work.

Name	Origin[a]	Marker[b]	Description
pTHSSd_1	ColE1	K/C	Splitposon in KanR ColE1 backbone
pTHSSd_2	ColE1	A	T7* RNAP 41-876 transposition target
pTHSSd_3	p15A*	S	T7* RNAP expression plasmid
pTHSSd_4	p15A*	S/K	P_{Tac} expression of T7 RNAP* split at 601
pTHSSd_5	p15A*	S/K	P_{Tac} expression of T7 RNAP* split at 601 with SZ17
pTHSSd_6	p15A*	S/K	P_{Tac} expression of T7 RNAP* split at 601 with SZ18
pTHSSd_7	p15A*	S/K	P_{Tac} expression of T7 RNAP* split at 601 with both SynZIPS
pTHSSd_8	pSC101	A	P_{T7} GFP reporter
pTHSSd_9	pSC101	A	P_{T3} GFP reporter
pTHSSd_10	pSC101	A	P_{K1F} GFP reporter
pTHSSd_11	pSC101	A	P_{CGG} GFP reporter
pTHSSd_12	pSC101	A	P_{T7} RFP reporter
pTHSSd_13	pSC101	A	reporter negative control
pTHSSd_14	p15A*	S	Triple split (at 67, 601-SZ)
pTHSSd_15	p15A*	S	Triple split no fragment 1:67
pTHSSd_16	p15A*	S	Triple split no fragment 67:601-SZ
pTHSSd_17	p15A*	S	Triple split no fragment SZ-601:883
pTHSSd_18	p15A*	S	Quad split (at 61, 179, 601-SZ)
pTHSSd_19	p15A*	S	Quad split no fragment 1:67
pTHSSd_20	p15A*	S	Quad split no fragment 67:179
pTHSSd_21	p15A*	S	Quad split no fragment 179:601-SZ
pTHSSd_22	p15A*	S	Quad split no fragment SZ-601:883
pTHSSd_23	p15A*	S	P_{Tac} T7 σ fragment expression
pTHSSd_24	p15A*	S	P_{Tac} T3 σ fragment expression
pTHSSd_25	p15A*	S	P_{Tac} K1FR σ fragment expression
pTHSSd_26	p15A*	S	P_{Tac} CGG σ fragment expression
pTHSSd_27	p15A*	S	P_{Tac} null fragment (σ_{CGG} Y639A) expression
pTHSSd_28	p15A*	S	P_{Tac} RFP expression
pTHSSd_29	p15A*	S	P_{Tac} α fragment expression
pTHSSd_30	p15A*	S	P_{Tac} GFP-α expression
pTHSSd_31	p15A*	S	P_{Tac} α-GFP expression
pTHSSd_32	p15A*	S	P_{Tac} RFP-α expression
pTHSSd_33	p15A*	S	P_{Tac} α-RFP expression
pTHSSd_34	p15A*	S	P_{Tac} GFP expression
pTHSSd_35	p15A*	S	inducible expression negative control v1
pTHSSd_36	p15A*	S	inducible expression negative control v2
pTHSSd_37	p15A*	S	Inducible full length T7* RNAP control
pTHSSd_38	BAC	K	High core fragment expression (high resource allocator)
pTHSSd_39	BAC	K	Low core fragment expression (low resource allocator)
pTHSSd_40	BAC	K	High core fragment expression without SynZIP
pTHSSd_41	BAC	K	High full length T7 RNAP* expression
pTHSSd_42	BAC	K	β core fragment expression
pTHSSd_43	BAC	K	core fragment expression negative control
pTHSSd_44	ColE1	C	constitutive expression of T7 σ fragment
pTHSSd_45	ColE1	C	constitutive expression of T3 σ fragment
pTHSSd_46	ColE1	C	constitutive expression of K1FR σ fragment
pTHSSd_47	ColE1	C	constitutive expression of CGG σ fragment
pTHSSd_48	ColE1	C	constitutive expression negative control
pTHSSd_49	p15A*	S	P_{Tac} T3 σ fragment, pTet K1FR σ fragment expression
pTHSSd_50	p15A*	S	P_{Tac} GFP, pTet RFP expression
pTHSSd_51	pSC101	A	pSC101 α fragment compensated reporter
pTHSSd_52	pUC19	A	pUC19 α fragment compensated reporter

a. *ColE1: derived from pSB1C3, p15A*: derived from pSB3C5, appears to maintain at a higher copy number than p15A, pSC101: derived from pUA66, BAC: derived from pBACr-Mgr940, pUC19: derived from pUC19.*

b. *A: ampicillin, K: kanamycin, C: chloramphenicol, S: spectinomycin.*

VII. References

Anderson JC, Voigt CA & Arkin AP (2007) Environmental signal integration by a modular AND gate. *Mol Syst Biol* **3:** 133

Bonner G, Patra D, Lafer EM & Sousa R (1992) Mutations in T7 RNA polymerase that support the proposal for a common polymerase active site structure. *EMBO J* **11:** 3767–3775

Chao H, Houston Michael E., Grothe S, Kay CM, O'Connor-McCourt M, Irvin RT & Hodges RS (1996) Kinetic Study on the Formation of a de Novo Designed Heterodimeric Coiled-Coil: Use of Surface Plasmon Resonance To Monitor the Association and Dissociation of Polypeptide Chains. *Biochemistry* **35:** 12175–12185

Cheetham GM & Steitz TA (1999) Structure of a transcribing T7 RNA polymerase initiation complex. *Science* **286:** 2305–2309

Dykxhoorn DM, St. Pierre R & Linn T (1996) A set of compatible tac promoter expression vectors. *Gene* **177:** 133–136

Goldhaber-Gordon I, Early MH, Gray MK & Baker TA (2002) Sequence and Positional Requirements for DNA Sites in a Mu Transpososome. *J Biol Chem* **277:** 7703–7712

Green B, Bouchier C, Fairhead C, Craig NL & Cormack BP (2012) Insertion site preference of Mu, Tn5, and Tn7 transposons. *Mob DNA* **3:** 3

Haapa S, Taira S, Heikkinen E & Savilahti H (1999) An efficient and accurate integration of mini-Mu transposons in vitro: a general methodology for functional genetic analysis and molecular biology applications. *Nucleic Acids Res* **27:** 2777–2784

Hoeller BM, Reiter B, Abad S, Graze I & Glieder A (2008) Random tag insertions by Transposon Integration mediated Mutagenesis (TIM). *J Microbiol Methods* **75:** 251–257

Jones D (2006) European Patent: EP1838851 - Polypeptide mutagenesis method.

Lee I & Harshey RM (2001) Importance of the conserved CA dinucleotide at mu termini. *J Mol Biol* **314:** 433–444

Lou C, Stanton B, Chen Y-J, Munsky B & Voigt CA (2012) Ribozyme-based insulator parts buffer synthetic circuits from genetic context. *Nat Biotechnol* **30:** 1137–1142

Mizuuchi M & Mizuuchi K (1993) Target Site Selection in Transposition of Phage Mu. *Cold Spring Harb Symp Quant Biol* **58:** 515–523

Mookhtiar KA, Peluso PS, Muller DK, Dunn JJ & Coleman JE (1991) Processivity of T7 RNA polymerase requires the C-terminal Phe882-Ala883-COO- or 'foot'. *Biochemistry* **30:** 6305–6313

Moon TS, Lou C, Tamsir A, Stanton BC & Voigt CA (2012) Genetic programs constructed from layered logic gates in single cells. *Nature* **491:** 249–253

Patrick WM, Firth AE & Blackburn JM (2003) User-friendly algorithms for estimating completeness and diversity in randomized protein-encoding libraries. *Protein Eng* **16:** 451–457

Pédelacq J-D, Cabantous S, Tran T, Terwilliger TC & Waldo GS (2006) Engineering and characterization of a superfolder green fluorescent protein. *Nat Biotechnol* **24:** 79–88

Pettersen EF, Goddard TD, Huang CC, Couch GS, Greenblatt DM, Meng EC & Ferrin TE (2004) UCSF Chimera—A visualization system for exploratory research and analysis. *J Comput Chem* **25:** 1605–1612

Poussu E, Jäntti J & Savilahti H (2005) A gene truncation strategy generating N- and C-terminal deletion variants of proteins for functional studies: mapping of the Sec1p binding domain in yeast Mso1p by a Mu in vitro transposition-based approach. *Nucleic Acids Res* **33:** e104–e104

Poussu E, Vihinen M, Paulin L & Savilahti H (2004) Probing the α-complementing domain of E. coli β-galactosidase with use of an insertional pentapeptide mutagenesis strategy based on Mu in vitro DNA transposition. *Proteins* **54:** 681–692

Ryan EP & Sontag ED (2006) Well-defined steady-state response does not imply CICS. *Syst Control Lett* **55:** 707–710

Salis HM, Mirsky EA & Voigt CA (2009) Automated design of synthetic ribosome binding sites to control protein expression. *Nat Biotechnol* **27:** 946–950

Shetty RP, Endy D & Knight TF (2008) Engineering BioBrick vectors from BioBrick parts. *J Biol Eng* **2:** 5

Temme K, Hill R, Segall-Shapiro TH, Moser F & Voigt CA (2012) Modular control of multiple pathways using engineered orthogonal T7 polymerases. *Nucleic Acids Res* **40:** 8773–8781

Yanisch-Perron C, Vieira J & Messing J (1985) Improved M13 phage cloning vectors and host strains: nucleotide sequences of the M13mpl8 and pUC19 vectors. *Gene* **33:** 103–119

Zaslaver A, Bren A, Ronen M, Itzkovitz S, Kikoin I, Shavit S, Liebermeister W, Surette MG & Alon U (2006) A comprehensive library of fluorescent transcriptional reporters for Escherichia coli. *Nat Methods* **3:** 623–628
